# Comprehension of the Presupposition Trigger *Ye* “Also” by Mandarin-Speaking Preschoolers With and Without Autism Spectrum Disorders

**DOI:** 10.3389/fpsyg.2020.570453

**Published:** 2020-10-28

**Authors:** Shasha An, Cory Bill, Qi Yang

**Affiliations:** ^1^Faculty of English Language and Culture, Center for Linguistics and Applied Linguistics, Guangdong University of Foreign Studies, Guangzhou, China; ^2^Department of Linguistics, Faculty of Humanities, University of Konstanz, Konstanz, Germany; ^3^Leibniz-Centre General Linguistics (ZAS), Berlin, Germany; ^4^Department of Philosophy, Tongji University, Shanghai, China

**Keywords:** Autism Spectrum Disorders, Mandarin-speaking preschoolers, linguistic impairments, presuppositions trigger, *ye* “also”

## Abstract

Individuals with autism spectrum disorders (ASD) have been reported to be widely impaired in their understanding of linguistic expressions that rely on elements of the context or norms of communication. The accurate interpretation of sentences conveying presuppositions often relies on such content, however, little previous research has investigated the ASD population’s understanding of these sentences. The present study attempts to remedy this by exploring the understanding that Mandarin-speaking preschoolers with ASD and their typically developing (TD) peers have of sentence containing the presupposition trigger *ye* “also”. We used a Picture Selection Task and found that Mandarin-speaking preschool children with ASD performed significantly worse than their TD peers with regard to their understanding of the presuppositional content of sentences containing this presupposition trigger. Additionally, in contrast with previous results, TD preschoolers’ understanding of this presupposition trigger was found to be adult-like. We attribute this to an improved experimental design.

## Introduction

Over the last few decades, a great many studies have revealed that individuals with autism spectrum disorder (ASD) are impaired in their ability to accurately comprehend certain types of linguistic meaning (Lord and Paul, 1997; Tager-Flusberg and Cooper, 1999; Tager-Flusberg, 2000; Loukusa and Moilanen, 2009; among many others). Notably, these tended to be types of linguistic meaning that rely on contextual/social knowledge to be accurately derived. For example, the ASD population has been reported to be impaired in comprehending figurative language (Norbury, 2005), as well as in understanding humor, irony, and jokes (Emerich et al., 2003; Martin and McDonald, 2004; Deliens et al., 2018).

Interestingly, a number of recent studies have found that there are a few types of linguistic meaning that individuals with ASD appear to understand as readily as their TD peers, despite them also being thought to rely on contextual/social knowledge. Specifically, the performance of people with ASD has been found to match their TD peers when it comes to accurately interpreting indirect requests and scalar implicatures (Pijnacker et al., 2009; Chevallier et al., 2010; Su and Su, 2015; Deliens et al., 2018). Considered together, these findings raise the possibility that the ability to understand language in context may only be selectively impaired in individuals with ASD.

The present study attempts to further investigate language impairments in the ASD population by focusing on their understanding of the presuppositions conveyed by certain lexical items (i.e., presupposition “triggers”). To understand how presupposition triggers contribute to linguistic meaning, a hearer has to be able to distinguish the presupposed information introduced by the trigger from any non-presupposed information (e.g., entailments, implicatures) and also understand how these various components interact and contribute to the final meaning of a host-utterance (Karttunen, 1974; Stalnaker, 1974, 2002; Levinson, 1983; Cheung et al., 2020).

In recent years, a small but growing number of acquisition studies have investigated presuppositions in TD populations (Berger and Höhle, 2012; Amaral and Cummins, 2015; Romoli et al., 2015; Schwarz, 2015; Bill et al., 2016). However, little attention has been paid to the understanding that individuals with ASD have of presuppositions, including how an understanding of them is acquired by children with ASD.

As far as we know, Cheung et al. (2017, 2020) are the only studies that have investigated the understanding that individuals with ASD have of presupposition triggers. Notably, these studies targeted Cantonese-speaking school-aged children and did not include the Cantonese counterpart of the presupposition trigger “also”. In other words, the current study is the first work to investigate the understanding that people with ASD have of the presupposition trigger “also”.

The presupposition trigger “also” is a focus particle, meaning that it associates with some other constituent in the sentence and its exact contribution to sentence meaning is determined by the set of alternatives in the discourse model. That is, in order to accurately understand a sentence containing the presupposition trigger “also”, one has to be able to identify and access the relevant shared information between the speaker and listener, i.e., from the linguistic context, the situational context, and world knowledge (Höhle et al., 2009). Given that deriving an accurate interpretation of such sentences relies on identifying which elements of the context are relevant, and given that this is an aspect of sentence interpretation that people with ASD have been shown to have difficulties with, it is plausible that this population might experience difficulties accurately understanding sentences containing this particle. As for previous work, not only have no existing studies investigated the understanding that preschoolers with ASD have of *ye* “also”, but no work has looked at the understanding that any individuals with ASD have of this trigger. Therefore, this investigation has the potential to provide important insights into the linguistic competence of children with ASD as well as the ASD population more generally.

## Literature Review

### Theoretical Background of Presuppositions

The presuppositions of a given sentence are the propositions that are required to be a part of the conversational common ground in order for that sentence to be felicitously uttered^1^ (Karttunen, 1974). To put it another way, in order for a given utterance to be felicitous, its presuppositions must be agreed to by all conversational participants. For example, the utterance in (1) conveys the presupposition that the speaker owns a hat; therefore, it can only be felicitously uttered in contexts where the fact that the speaker owns a hat is already part of the common ground.

(1) My hat is yellow.

According to a traditional analysis (Karttunen, 1974; Stalnaker, 1974), when (1) is uttered in a context in which the identified presupposition is not a part of the common ground, there are two possible outcomes. The first and arguably most common outcome is that a hearer “accommodates” the presupposition and interprets the sentence as though the presupposition had already been a part of the common ground. The second possible outcome is for “presupposition failure” to occur. This will result in the utterance being perceived by the hearer as infelicitous and lacking a truth value (i.e., being “undefined”), and moreover, the propositions conveyed by the utterance will not be added to the common ground (Karttunen, 1974; Stalnaker, 1974; Heim, 1983).

The lexical items or phrases that engender presuppositions are called “presupposition triggers” (Levinson, 1983; Huang, 2014). As mentioned, one such presupposition trigger is the focus particle, or more precisely the additive particle, “also”. The inclusion of “also” in a sentence results in the meaning of its host-sentence being partitioned into an asserted element and a presupposed element (Krifka, 1998; Lee, 2002). Specifically, the additive particle “also” targets a focused constituent within its host-sentence and conveys the “existential presupposition” that the predicate holds for at least one alternative of the element in focus. For example, in the case of a sentence like (2a), if “tennis” is focused, then the sentence presupposes that Mary played something other than tennis, as shown in (2c). In contrast, if “Mary” is focused, then the sentence presupposes that someone else played tennis, as shown in (2d). The assertion conveyed by the sentence in (2a) is always (2b).

(2) a. Mary also played tennis.
b. Assertion: Mary played tennis.
c. Presupposition: There is an x ≠ tennis, and Mary played x.
d. Presupposition: There is an x ≠ Mary, and x played tennis.

The counterpart of the additive particle “also” in Mandarin Chinese, namely *ye*, partitions sentence meanings into the same presuppositions and assertions (Liu, 2009; Liu et al., 2011; Wang, 2011; Ji, 2015). Taking an example from Liu et al. (2011) to illustrate:


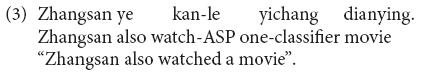


As with “also”, the focus particle y*e* in Mandarin Chinese can be associated with the subject or the verbal phrases in the sentence, with each association giving rise to different presuppositions. More specifically, when the subject “Zhangsan” is emphasized, the sentence in (3) presupposes that someone other than Zhangsan watched a movie; when stress is put on the verbal phrase *kanle yichang dianying* “watched a movie”, the sentence in (3) presupposes that Zhangsan did something else, in addition to watching a movie (Liu et al., 2011).

Notably, in order to accurately identify the contribution of the additive particle to the final meaning of a sentence, the hearer must identify the focused constituent in the utterance, as well as the set of relevant alternatives in the common ground. This process requires, among other things, integrating information from different sources. For example, as shown in (4), it often involves the integration of prosodic content as well as content from the immediate situational context. That is, given the context in (4), deriving the correct presuppositions for the sentences in (4a) and (4b) requires an accurate perception of the prosodic contours of the utterance in order to identify which element is the focused constituent. Moreover, in (4) the presuppositions associated with (4a) and (4b) are satisfied, not from the previous linguistic discourse, but from the immediate situational context.

(4) Anne and Bill are attending a work party. They run into each other out the front of the building where the party is being held. Anne is holding a packet of chips and a bottle of wine. Bill is only holding a packet of chips, but he also has a bottle of wine in his backpack.
  (4a) Bill: Oh hi Anne. [I]_*F*_ also brought a bottle of wine.
  Presupposition: Someone else (i.e., Anne) brought a bottle of wine.
  (4b) Bill: Oh hi Anne, I also brought [a bottle of wine]_*F*_.
  Presupposition: Bill brought something else (i.e., a packet of chips).

In this way, determining both the nature of the presuppositions conveyed by (4a) and (4b), as well as whether they are satisfied in the context, requires integrating information from different sources (i.e., from the sentence prosody and from the situational context).

### Acquisition of “Also”

Previous studies (Lee, 2002; Nederstigt, 2003; Hüttner et al., 2004; Bergsma, 2006; Matsuoka et al., 2006; Höhle et al., 2009; Müller et al., 2009; Liu et al., 2011, among others) have reported that TD children start producing the focus particle “also” as early as one and a half years of age across a variety of languages, including Cantonese, Mandarin, Japanese, Dutch, and German. However, a series of studies have also found that TD children’s comprehension of this particle is not adult-like until around 8 years of age. More specifically, before this age, TD children do not appear to accurately understand the presuppositional content introduced by “also” (for relevant reviews, see Höhle et al., 2009; Berger and Höhle, 2012).

It is worth noting, however, that many of these previous comprehension-based studies adopted Picture Selection/Judgment tasks, which presented test sentences, like (5), in isolated, “out of the blue” contexts, that is, with no/minimal introduction or leading sentences.

(5) The boy is also patting a dog.

Following this, participants were required to select a matching picture or to judge whether the presented picture matched the utterance (Hüttner et al., 2004; Bergsma, 2006; Matsuoka et al., 2006; Höhle et al., 2009; Liu et al., 2011). For example, Liu et al. (2011) presented a picture where a boy and a girl are petting a dog and a cat, respectively, and asked participants (4;02–7;10) to judge whether the sentence in (5) was a correct description of the picture. The results of this study suggested that children could not access the presupposed meaning of “also” in an adult-like fashion until around 7 or 8 years of age (7;02–7;10, M = 7;05). Specifically, the participants of a 7-year-old group could interpret “also” in an adult-like manner at a rate of 86%, while the 6-year-old group (6;00–6;11, M = 6;06) only interpreted it in this manner at a rate of 34%. A similar result was found in Bergsma (2006), which adopted a Picture Selection Task, where participants were presented with three pictures and asked to select which of them matched the presented single test sentence. The study found that 6–7 years of age Dutch-speaking children (6;05–7;11) were not adult-like in their understanding of *ook* “also”. Finally, a study with the same experimental design, Hüttner et al. (2004), found that German-speaking children (5;1–7;8, M = 5;8) did not access an adult-like interpretation of *auch* “also”.

One thing these previous studies have in common is that they presented the test sentences in quite an isolated and unnatural manner, which is very different from the way they would be presented in a “normal” discourse. For example, in a typical conversation, an utterance like (5) would often be produced after there had either been some mention of the fact that someone else had patted “a dog”, or some mention of “the boy” having done something else, that is, after the content presupposed by the additive particle had been clearly added to the common ground.

In contrast, many of the studies investigating TD children’s understanding of the additive particle (e.g., Hüttner et al., 2004) presented the test sentence without any (or with minimal) preceding discourse. Following such a presentation, children were required to identify which picture (from a set) was accurately described by the presented utterance. Presenting the test sentences in this isolated manner means that in order to get the “correct” interpretation, not only must a participant understand the presuppositional content conveyed by the additive particle, but they must detect that some of the presented pictures depict a presupposition failure and reject them on that basis. Previous work has found that even adults often fail to reject pictures on this basis, seemingly due to a tendency to only focus on whether the picture satisfies the truth conditions of a target sentence (Hornby, 1974; Kim, 2008). Given the difficulties that adults have “successfully” completing such a task, it seems likely that the use of similar tasks in acquisition research may have resulted in an under-estimation of children’s understanding of the presuppositional content of these particles (Höhle et al., 2009; Berger and Höhle, 2012).

In fact, this interpretation of the previous literature is supported by a more recent study by Berger and Höhle (2012), which tested TD children’s understanding of additive particles using a method that was free from the issues just outlined. That is, they used a paradigm in which the relevant presuppositions were explicitly satisfied in the context, but where a knowledge of them was still required to give the target response. Berger and Höhle (2012) found that such changes improved preschoolers’ performance in displaying an accurate interpretation of “also”, such that it was more adult-like than had been found in previous studies. Following Berger and Höhle (2012), the present study tried to give children the best chance of interpreting “also” appropriately by increasing the salience of the elements satisfying the presupposition and making the presuppositional content clearly relevant to the successful completion of the experimental task.

In contrast to the large numbers of studies which have investigated “also” in TD children, to the best of our knowledge, no existing research has investigated atypical children’s understanding of this presupposition trigger. That is, Cheung et al. (2017, 2020) (which we will turn to next) are the only existing work that has examined the interpretations of presupposition triggers in children with ASD, and they did not include this trigger in their investigation.

Both Cheung et al. (2017, 2020) adopted the same task, which involved participants judging whether a given utterance was or was not a presupposition of a preceding utterance. Specifically, participants were presented with Cantonese versions of sentences like (6a), followed up with either the continuation in (6b) or (6c). The sentence in (6a) conveys the presupposition that “Daaiman has a sister”. Therefore, participants were considered to have understood the presupposition if they judged (6b) as “correct” and (6c) as “incorrect”.

(6) a. Daaiman’s sister will be 10 years old next year. That is to say,
b. Daaiman has a sister.
c. Daaiman doesn’t have a sister.

Cheung et al. (2017) investigated children with ASD’s understanding of a range of presuppositions and compared them with their Typically Developing (TD) peers. The participants with ASD ranged in age from 6;06 to 14;03 (M = 8;09). Cheung et al. (2017) found evidence that children with ASD’s understanding of existential (definite descriptions, proper names and possessives), factive (factive verbs), lexical (change-of-state verbs and iteratives), and structural (cleft sentences and temporal clauses) presuppositions was worse than their age-matched TD peers. One limitation of this study was that it did not control for participants’ language ability, a variable that is likely to have an influence on participants’ performance in this task. Another limitation of the study was that they grouped together a number of presupposition triggers that might have independent developmental trajectories (e.g., change-of-state verbs and iteratives were grouped together as “lexical presuppositions”). That is, it is possible that there is some variation between the triggers that Cheung et al. (2017) grouped together with regard to the ease with which an understanding of their presuppositional content is acquired. For this reason, we think a natural next step for research in this area is to investigate some of these triggers in isolation, thereby allowing us to check whether, as far as acquisition is concerned, they actually do follow the same trajectory.

Cheung et al. (2020) investigated the understanding that Cantonese-speaking children with ASD (7;07–11;11, M = 9;01) have of the presuppositions associated with seven different types of presupposition triggers, namely, definite descriptions (“the professor”), factive predicates (“know”, “regret”), change-of-state verbs (“start”, “quit”), implicative verbs (“forget”), iteratives (“again”, “not anymore”), counterfactual conditionals (“if”), and temporal clauses (“before”). It was found that children with ASD performed significantly worse than their age-matched TD peers, but similarly to their language-matched TD counterparts, in regard to their understanding of the presuppositions associated with all of the trigger classes, except for temporal clauses. That is, in the case of the presuppositions triggered by temporal clause, Cheung et al. (2020) found that Cantonese-speaking children with ASD showed a deficit in their understanding even when age, language ability, and non-verbal intelligence were controlled for. In sum, Cheung et al. (2017, 2020) show that while children with ASD’s understanding of presuppositions is below their age-matched peers, it is generally similar to their language-matched peers (with the exception of temporal clauses).

One thing worth noting at this point is that (as we have already discussed to some extent) research on TD children’s understanding of presuppositions has found substantial variation in children’s performance between methodologies and between triggers (e.g., Höhle et al., 2009; Berger and Höhle, 2012). Moreover, as Cheung et al. (2017, 2020) demonstrate, when related variables (for instance, language ability) are included and controlled for, a more complete developmental picture of the ASD population’s understanding of presuppositions is revealed. Therefore, some promising avenues for further investigations in this area include exploring new triggers, using different methods, and controlling for as many influential variables as possible.

The present study contributes to this effort by using a novel method to examine Mandarin-speaking children with ASD’s understanding of the presuppositions associated with sentences containing the trigger *ye* “also”. This trigger has not been included in any previous investigations of the ASD population, as far as we are aware. Moreover, existing research that studied the acquisition of the presupposition trigger “also” in TD children reported mixed findings, which may have been caused by differences in the experimental methods. The unique experimental design in this study is expected to provide a better chance for children to demonstrate their knowledge of the presupposition trigger “also”. Furthermore, the present study targets Mandarin-speaking preschoolers with and without ASD, whose age, receptive language ability, intelligence, working memory, inference ability, and executive function will be measured and controlled for.

### Research Aims

This study’s primary aim is to further investigate the understanding that children with ASD and individuals with ASD more broadly have of presuppositions. The current study does this by testing how participants from this population interpret sentences containing the presupposition trigger *ye* “also”. Moreover, as noted above, the experimental design in previous acquisition studies, which used isolated sentences as test stimuli, might not have provided a felicitous context for the production and comprehension of this trigger, and thus, may not represent an accurate picture of TD children’s knowledge of “also”. In an effort to improve the experimental design and give children the best chance of accessing the adult-like interpretation of “also”, this study presented the target sentences in contexts where the relevant presuppositions were plausibly satisfied in the preceding clause, and where accessing the presupposition was integral to successfully completing the task. Presenting the stimuli in this manner allows us to address a secondary research aim: to test whether TD preschoolers are capable of interpreting the presupposition trigger “also” in the same way as adults, when the salience of the elements satisfying the presupposition is increased and the overall felicity of the utterance within the experimental context is improved.

## Materials and Methods

### Participants and Procedures

Twenty-five participants with ASD were recruited from a training school for preschoolers with ASD. All of the children who were admitted to the training school were required to provide formal diagnosis results. The ASD participants in the present study had been diagnosed by experienced child psychiatrists or child neurologists as meeting the criteria of the *Diagnostic and Statistical Manual of Mental Disorders, Fifth Edition* (*DSM-5*; American Psychiatric Association, 2013) and the Chinese version of the Autism Spectrum Quotient: Children’s Version (AQ-Child; Auyeung et al., 2008) for ASD. The children had been recorded as demonstrating mild to moderate degrees of qualitative impairments in social interactions and communications, as well as restricted, repetitive, and stereotyped interests and activities.

Based on reports from their families and teachers, all of the participants with ASD did not have any other neuropsychiatric or developmental disorders, or any hearing loss or language impairments. The diagnosis of each participant was confirmed by a family member. Taking these facts into account, we decided not to carry out another diagnosis interview or evaluation to confirm their ASD symptomatology.

Twenty-two TD preschoolers and college students were recruited from a mainstream kindergarten and a university, respectively. All of the TD participants had not been reported as having any developmental or psychiatric disorders, learning disabilities, or language impairments.

The children were administered the Chinese Peabody Picture Vocabulary Test (C-PPVT; Sung and Miao, 1990) to assess their receptive language ability. Their Verbal Intelligence, Non-Verbal Intelligence and Full Intelligence, Inference Ability and Working Memory were evaluated using the Chinese version of the Wechsler Preschool and Primary Scale of Intelligence-Fourth Edition (C-WPPSI-IV; Li and Zhu, 2014). Their executive function was assessed with the Day/Night Stroop Task (Gerstadt et al., 1994) and the Flexible Item Selection Task (Jacques and Zelazo, 2001). Three children with ASD were excluded from the study due to attention problems. Table 1 presents descriptive characteristics of the participants included in the present study^2^.

**TABLE 1:** Characteristics of participants.

	**ASD-children**	**TD-children**	**TD-adults**
Number of participants	22	22	22
Age in years	5.24 (0.49)	5.24 (0.31)	19.32 (0.84)
Age range in years	4.30–6.09	4.51–5.58	18–20
C-PPVT	118.18 (18.19)	116.68 (9.12)	–
Verbal intelligence (VIQ)	115.18 (18.72)	110.82 (13.50)	–
Non-verbal intelligence (N-VIQ)	114.18 (18.10)	110.14 (11.36)	–
Full intelligence (FIQ)	115.41 (18.76)	110.41 (10.31)	–
Inference ability (IA)	113.55 (14.11)	109.95 (13.18)	–
Working memory (WM)	106.82 (22.53)	100.45 (11.87)	–
Executive function-stroop task	0.61 (0.34)	0.67 (0.31)	–
Executive function-FIST	0.67 (0.35)	0.76 (0.29)	–

*Numbers presented are group means, with standard deviation shown in parentheses.*

All of the participants were introduced to the task and tested individually. The experiment was conducted in quiet rooms in the schools. Child participants and their family as well as adult participants were fully informed about the procedures. Written consent was obtained from adult participants and from the family of the child participants. In addition, oral consent for taking part in the study was given by each child participant.

The procedures were in accordance with the ethical guidelines presented in the Declaration of Helsinki (World Medical Association General Assembly, 1964) and its later amendments or comparable ethical standards.

### Experimental Design

We used the Picture Selection Task paradigm. The main part of the experiment was comprised of 10 filler trials and 10 test trials, which were interspersed with each other. All of the trials were compound sentences, which were made up of two simple clauses and recorded by a female Mandarin native speaker at a moderate speed. After recording, the verb phrases of the first clauses were replaced by sounds of “cars passing by”. As mentioned earlier, the presupposition introduced by the additive particle targets a focused constituent of its host-sentence and dictates that this constituent be interpreted in relation to a set of alternatives.

Specifically, the particle presupposes an “additive” relationship between the focused particle and its alternative/s. For example, if the subject “Jim” is focused in a sentence like “Jim also bought an apple”, then this sentence presupposes that someone else bought an apple. Therefore, a natural context in which to present such a sentence would be “Mary bought an apple; Jim also bought an apple”. Therefore, in our test trials, the first clause of the target sentence provides the alternative that satisfies the presupposition of the second clause, which contains the focus particle. That is, the presupposition trigger “also”, introduced in the second clause, will be interpreted in a context that already includes the first clause (Schwarz, 2015).

According to Stalnaker (1974, 2002) and Berger and Höhle (2012), introducing the focus particle in a discourse context where the content satisfying the relevant presupposition is absent or is of low salience would require the participant to carry out some extra step (i.e., accommodation or a content search) to access the target interpretation, and so may lead to an underestimation of children’s knowledge of this particle. This study attempted to avoid this by presenting “also” with a preceding clause, which plausibly satisfied the presupposition and so provided a felicitous context for the production of a sentence including “also”.

Four practice trials were given prior to the presentation of any test items to familiarize participants with the task. The experiment was introduced with the Mandarin equivalent of the following dialogue:

Hello. You are going to see many pictures, which are numbered 1, 2, 3, 4 (an experimenter pointing to the numbers), and hear many sentences. In each sentence, there is a part you cannot hear because there are cars passing by. Your job is to figure out what the unheard parts are and find the corresponding pictures for the sentences. When you find the correct pictures, you may tell me its number or point it out.

The practice trials^3^ were of the same structures and presented similarly to the trials in the main part of the experiment, but with the verb phrases in the second clause being replaced by the sounds of “cars passing by”. This was to make sure that participants were capable of processing compound sentences that were made up of two simple clauses. Corrective feedback was given to participants when they chose the wrong pictures.


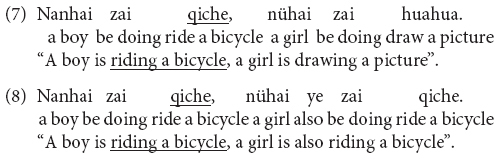


**FIGURE 1:**
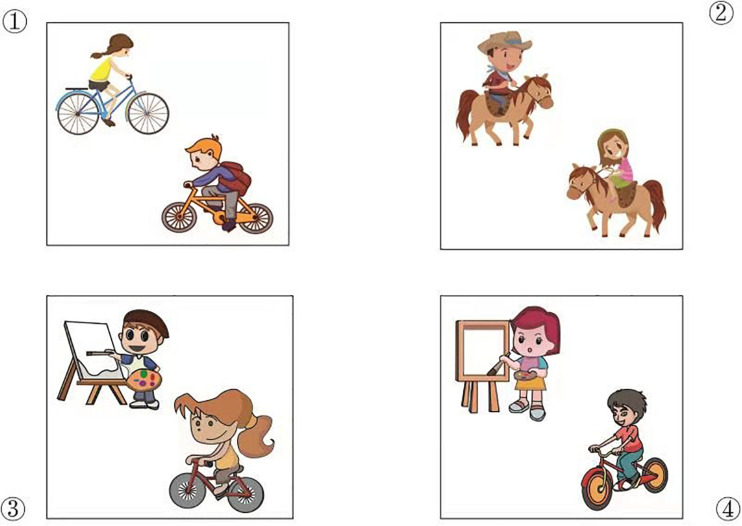
Pictures of example trials.

In the main part, participants were presented with filler and test trials, as exemplified in (7) and (8), in which the verb phrases of the first clauses (i.e., underlined sections) were made inaudible by playing sounds of passing cars. Figure 1 shows the pictures that were paired with these sentences. Upon hearing the filler sentence in (7), participants were expected to look for a picture where a girl is drawing a picture (i.e., Figure 1, picture ④). If a participant had acquired an understanding of the presuppositional content of the test sentence in (8), they were expected to select the picture in which a girl is riding a bicycle and a boy is carrying out the same activity (i.e., Figure 1, picture ①). On the other hand, if a participant had not acquired an understanding of the presuppositional content of the test sentence, they could interpret the second clause as just conveying that a girl is riding a bicycle, the same interpretation as the clause without *also*. In this case, picture ③ is a possible answer, in addition to picture ①. We coded responses that correctly selected picture ① as *also-correct* and responses that selected picture ③ as *also-without-presupposition*. Answers as ② or ④ were coded as *also-false*. The positions of the target pictures were counterbalanced.

## Results

Table 2 presents the mean and standard deviation of the answers provided by each group. We analyzed the data with the software package of SPSS 25. Each group correctly answered the filler trials over 96% of the time, which suggests that all of the participants understood the task and were capable of processing sentences where the first clause is partially “obscured”.

**TABLE 2:** Proportion of answers of participants.

**Answers**	**ASD (*n* = 22)**	**TD-children (*n* = 22)**	**TD-adults (*n* = 22)**
Also-correct	0.67 (0.35)	0.93 (0.09)	0.97 (0.06)
Also-without-presupposition	0.31 (0.35)	0.05 (0.07)	0.03 (0.06)
Also-false	0.02 (0.06)	0.02 (0.05)	0.00 (0.00)

*Numbers presented in the table are means of each group, with standard deviation in parentheses.*

Participants’ answers to the test trials were evaluated by conducting a one-way ANOVA, with the adoption of Tamhane’s T2 test for *post hoc* pairwise multiple comparisons. The ANOVA revealed significant differences among the three groups with regard to *also-correct* answers, *F*(2, 63) = 12.53, *p* = 0.000, η^2^ = 0.28, and *also-without-presupposition* answers, *F*(2, 63) = 11.95, *p* = 0.000, η^2^ = 0.28. No significant difference was found for *also-false* answers, *F*(2, 63) = 1.53, *p* = 0.23, η^2^ = 0.04.

Specifically, ASD children chose significantly less *also-correct* answers (*p* = 0.01) and more *also-without-presupposition* answers (*p* = 0.009) than TD children. Both groups performed at a similar level in their choices of *also-false* answers (*p* = 0.99). Moreover, to control for the effect of age, receptive language ability, non-verbal intelligence, verbal intelligence, working memory, inference ability, and executive functions, we further compare the performance of both groups with a general linear model. The above-mentioned potential influential variables were treated as covariances. After controlling for the effect of the variables, we found that TD children provided significantly more *also-correct* answers than ASD children group (*p* = 0.006). Moreover, ASD group chose significantly more *also-without-presupposition* answers than TD group (*p* = 0.004). Both groups performed similarly with regard to their choice of *also-false* answers (*p* = 0.97).

Finally, TD children and adults performed very similarly. That is, we found no difference between the two groups with regard to their choices of *also-correct* answers (*p* = 0.16), *also-without-presupposition* answers (*p* = 0.48), and *also-false* answers (*p* = 0.28).

## Discussion

The present study set out to further our understanding of the linguistic competence of ASD individuals by exploring ASD children’s understanding of the additive particle. We primarily examined whether Mandarin-speaking preschoolers with ASD had the same level of knowledge of the presuppositional content associated with sentences containing the presupposition trigger *ye* “also” as their TD peers. A secondary goal of this experiment was to investigate whether TD preschool children would readily access an adult-like interpretation of sentences containing this presupposition trigger.

### Methodological Implications

As for the second goal, our experiment found no difference in the interpretations of our test sentences accessed by TD adults and TD children. This finding differs from the series of previous studies (Hüttner et al., 2004; Bergsma, 2006; Matsuoka et al., 2006; Höhle et al., 2009; Liu et al., 2011) that found preschool children were not adult-like in their understanding of the presupposed meaning of “also”. In contrast, it is in line with previous work showing that children’s understanding of the presuppositions conveyed by sentences including focus particles like “also” is improved when presented in a more felicitous context (Berger and Höhle, 2012). We therefore interpret our result as indicating that, as intended, our experiment presented participants with a similarly felicitous context. That is, it seems likely that by increasing the salience of the elements of the context that satisfied the relevant presuppositions, we enabled TD Mandarin-speaking preschool children (4.3–6.09 years old, M = 5.24) to achieve adult-like competence in their understanding of sentences containing the presupposition trigger *ye*. To put it another way, presenting our test sentences in a context where the content satisfying its presupposition was presented in the immediately preceding clause reduced the ancillary demands associated with accessing an adult-like interpretation of these sentences. Therefore, we take our results to be a more accurate representation of TD children’s understanding of the presuppositional content of sentences containing the presupposition trigger “also”.^4^

This aspect of our results seems to support the argument, proposed by Crain and Fodor (1993), that it is only when appropriate discourse conditions are provided that children’s true understanding of linguistic phenomena is revealed. In the case of presuppositions, when an utterance conveying a presupposition is presented in isolation, with none or very little of the surrounding context in which such an utterance would usually be produced, children’s (and in fact, even adults’) performance in understanding that utterance can often be underestimated. Therefore, we take our results as providing further support for Berger and Höhle (2012)’s suggestion that these factors of the experimental design should be given careful consideration when investigating children’s understanding of any presuppositional content.

### Theoretical Implications

The main novel contribution of this study was the finding that preschoolers with ASD demonstrated impairments in their understanding of the existential presupposition associated with the additive particle *ye* “also”, even after controlling for the effects of age, language ability, verbal intelligence, working memory, inference ability, and executive function. This study is the first investigation of ASD children’s (or of any individual with ASD’s) understanding of this specific presupposition trigger and this result identifies another respect in which the linguistic abilities of people with ASD appear to be impaired. This naturally raises the question of why children with ASD’s understanding of the presuppositional content of sentences containing “also” should be worse than that of TD children.

As we mentioned in the Literature Review section, accessing an adult-like interpretation of a sentence containing the focus particle “also” involves identifying the focused constituent within the host-sentence and picking out the set of alternatives related to this focused constituent from the discourse context. The successful identification of these elements and the derivation of the associated presupposition can involve, among other things, the integration of information from different sources. For example, our experiment was designed so that participants would be facilitated in achieving an accurate understanding of the presuppositional content of our test sentences if they understood the relationship between them and the preceding clauses. That is, if they took note of the mentioning of “a boy” in the first clause of (7), this should facilitate the accurate identification that the focused constituent in the second clause was “a girl” and not “riding a bicycle”. This aspect of our test sentences is notable because it has been suggested that when processing information, individuals with ASD demonstrate a unique processing style called “Weak Central Coherence” (WCC; Happé and Frith, 2006), which makes them more likely to focus on details and pay preferential attention to parts rather than wholes. It is possible that our results could be accounted for as being a result of such a WCC processing style. That is, WCC may have made it more difficult for children with ASD to accomplish the necessary step of identifying and integrating relevant information from multiple information sources, in order to accurately interpret sentences containing the presuppositional trigger “also”. Specifically, unlike TD children, they may have struggled to identify and/or integrate into their processing of sentence meaning, the prosodic information signaling the focused constituent and/or the fact that the preceding clause presented a plausible alternative to that focused constituent. We should note that many previous linguistic impairments in the ASD population have been accounted for by suggesting that this population have certain limitations that affect their accurate understanding of aspects of language associated with syntactic ability and vocabulary (Norbury, 2004, 2005), and Theory of Mind (Cummings, 2013). We remain open to alternative explanations of our results along these lines (or others). Future work in this area would benefit from the inclusion of tests measuring the syntactic, vocabulary, theory of mind and central coherence abilities of the participants in order to explore their role in presuppositions understanding.

Another point we should discuss is a certain contrast between our results and those obtained by Cheung et al. (2020). Our study investigated Mandarin-speaking preschoolers (4.30–6.09 years of age, M = 5.24) with ASD’s understanding of the presuppositional content of sentences containing the presupposition trigger *ye* “also”, which is a lexical presupposition trigger (Huang, 2014). Notably, Cheung et al. (2020) also investigated the interpretation of lexical presupposition triggers (i.e., definite descriptions, factive predicates, change-of-state verbs, implicative verbs and iteratives) in Cantonese-speaking school-aged children (7.58–11.92 years old, M = 9.07) with ASD. However, unlike us, Cheung et al. (2020) found that their ASD participants performed similarly with language-matched TD peers in their comprehension of the lexical presupposition triggers they investigated. This naturally raises the question of what factors might be responsible for the difference in results between our and this previous studies.

One possibility is that differences in matching criteria between our study and Cheung et al. (2020) are responsible for these different findings. Specifically, we assessed receptive language abilities using the Chinese Peabody Picture Vocabulary Test (C-PPVT test), the only available standardized assessment tool for receptive language ability in Mandarin Chinese. Notably, this test is less challenging than the textual comprehension test (TCT) used by Cheung et al. (2020), and so the language abilities of the children with ASD in the current study may have been overestimated. Additionally, the fact that we used a different assessment for Non-Verbal Intelligence from Cheung et al. (2020) might also have contributed to the different performances of participants with ASD. Future research could provide more comparable findings by using the same batteries of assessments to compare the developmental trajectories of different lexical presuppositions in children with ASD.

Finally, this study provides further evidence of difficulties that individuals with ASD have in understanding elements of linguistic meaning that rely on contextual/social knowledge or abilities. However, it is interesting to note that quite different results have been found in recent work investigating people with ASD’s understanding of another type of linguistic meaning that has been suggested to involve similar knowledge and abilities, namely, “scalar implicatures”. Specifically, studies by Pijnacker et al. (2009) and Chevallier et al. (2010) found that the understanding ASD participants displayed of this element of meaning was in line with their TD peers. Considered together, our results and the previous literature could be interpreted as evidence that people with ASD are selectively impaired when it comes to understanding elements of linguistic meaning that rely on identifying and integrating relevant elements of the context. This is an interesting prospect as it may help in identifying more precisely the impairments that people with ASD have. For example, Andrés-Roqueta and Katsos (2017) propose that the set of processes traditionally grouped under “pragmatics” should be re-grouped into two categories called “linguistic-pragmatics” and “social pragmatics”. In this sense, research investigating the understanding that ASD individuals have of linguistic meaning phenomena, with measures of various cognitive and linguistic abilities, promises to shed light not only on the linguistic abilities of this population but also on the nature of the targeted phenomena.

## Conclusion

The current study aimed to advance the knowledge of linguistic impairments in ASD people by examining how Mandarin-speaking preschoolers with ASD interpreted sentences containing the presupposition trigger *ye* “also”. We found that children with ASD performed significantly worse than their TD peers in understanding the presuppositional content of sentences including this trigger. We further found that TD 4–6-year-old children’s interpretation of this trigger is adult-like.

## Data Availability Statement

All datasets presented in this study are included in the article/Supplementary Material.

## Ethics Statement

The studies involving human participants were reviewed and approved by the Guangdong University of Foreign Studies. Written informed consent to participate in this study was provided by the participants’ legal guardian/next of kin.

## Author Contributions

SA conceived the study, designed, carried out the experiments, and drafted the manuscript. SA analyzed the data with the assistance of QY. SA and QY interpreted the data and discussed the structures of writing with each other. CB contributed to the writing and revision of the Introduction, Literature Review, Discussion, and Conclusion part of the manuscript. All authors contributed to manuscript revision, read, and approved the submitted version.

## Conflict of Interest

The authors declare that the research was conducted in the absence of any commercial or financial relationships that could be construed as a potential conflict of interest.

## Footnotes

1 The “common ground” is comprised of the set of propositions that all participants in a conversation have agreed to (Stalnaker, 2002).

2 As noted by a reviewer, we did not conduct a test directly measuring participants’ syntactic abilities, which is a parameter that could influence children’s ability to accurately interpret a sentence containing presuppositional content. We agree that this is a relevant parameter. However, there is (to our knowledge) no existing well-developed standardized test to assess syntactic ability in Mandarin Chinese; therefore, obtaining such a measurement was not straightforward. Having said that, we agree that research in this area would ideally include such a measure and so must accept its absence as a limitation of our study.

3 For example, one practice trial is as follows:

4 As noted by a reviewer, it is possible that our participants would have performed similarly to adults, even if our methodology had placed higher demands on participants (e.g., by presenting sentences with minimal/no surrounding context). While we cannot rule out the possibility that our TD child participants would have performed just as well with another methodology, we would again note that in many of the previous studies, which had such higher requirements, preschool age children were not adult-like in their performance (e.g., Hüttner et al., 2004). We take this as at least suggestive evidence that the noted properties of our methodology contributed to the relatively high performance children exhibited in our experiment.
